# Thermal Storage Properties of Molten Nitrate Salt-Based Nanofluids with Graphene Nanoplatelets

**DOI:** 10.1186/s11671-016-1519-1

**Published:** 2016-06-21

**Authors:** Qiangzhi Xie, Qunzhi Zhu, Yan Li

**Affiliations:** College of Energy and Mechanical Engineering, Shanghai University of Electric Power, No. 2103 Pingliang Road, Yangpu District, 200090 Shanghai China

**Keywords:** Thermal storage properties, Specific heat capacity, Graphene nanoplatelets, Solar salt, DSC, SEM

## Abstract

In this study, the effect of concentration of nanoparticles on the thermal storage properties of molten nitrate salt-based nanofluids with graphene nanoplatelets (GNPs) was investigated. Solar salt consisting of sodium nitrate and potassium nitrate was utilized as the base material for the nanofluids. Homogeneous dispersion of GNPs within the solar salt was observed through scanning electron microscopy analysis. For both solar salt and resultant nanofluids, differential scanning calorimetry was employed to measure the thermal storage properties, including characteristic temperatures of phase change, startup heat, and specific heat capacity (SHC). A maximum increase of 16.7 % in SHC at the liquid phase was found at an optimal concentration of 1 wt% of GNPs. At the same concentration, the onset temperature decreased by 10.4 °C, the endset temperature decreased by 4.7 °C, and the startup heat decreased by 9 %.

## Background

For solar thermal power generation technology, two major drawbacks related to solar energy should be solved. One is low energy density, and the other is poor continuity. Solar concentration technology is an effective means to solve the former, whereas thermal energy storage technology is a suitable method for the latter. Contemporary concentrated solar power plants utilize solar salt (binary nitrate salt mixtures) as a sensible thermal energy storage medium. Such power plants also use heat transfer fluid in some cases. Solar salt has several advantages [1]. First, solar salt is stable, nonflammable, nonexplosive, and nontoxic. Second, solar salt can stably operate at a temperature range of 290–600 °C. Lastly, within the operation temperature range, the thermal storage system for liquid solar salt can work at atmospheric pressure or slightly positive pressure, leading to convenient daily operations.

The thermophysical properties of solar salt are important to the efficiency and safety of thermal storage systems in power plants. The most important thermal storage properties are specific heat capacity (SHC), melting temperature, and startup heat. The volume and total cost of a storage system can decrease if molten salt with a high value of SHC is utilized [2]. The melting temperature of molten salt determines the low limit of the operation temperature in a thermal storage system. Meanwhile, if the melting point is high, then the temperature difference between the thermal storage material and the surrounding would be large. This phenomenon can result in increased heat dissipation and thus requires highly efficient thermal insulation [3]. With regard to startup heat, a large amount of heat is required to heat solar salt for solid-to-liquid phase transformation during the startup of a solar thermal power plant. Excessive startup heat slows down the melting process and increases the risk of local over-heating of molten salt [1].

Nanotechnology has been applied to develop a new working medium for different applications, such as heat transfer and heat storage. The stable colloidal suspensions of nanoscale particles inside various liquids are called “nanofluids” [4]. The effects of concentration on the SHC of nanofluids have been investigated extensively. Adding nanoparticles (NPs) to base fluids can generally increase the SHCs. Starace et al. experimentally investigated the SHC of 0.1 and 0.5 wt% AlN NPs with a mineral oil mixture and found that SHC was enhanced by 2 and 3 %, respectively [5]. However, decrements in the SHC of nanofluids have been observed by researchers. Choi and Zhang reported that the SHC of water decreased by 7.6–25 % after adding 2.5–10 vol% of 50 nm Al_2_O_3_ NPs [6]. Starace et al. observed that the changes in the heat capacity of the nanofluids based on the mixtures of poly-α olefin and chloroform varied from 5 % increase to 10 % degradation after adding 0.1–2.3 wt% of 15 nm Fe and Fe_3_O_4_ NPs, respectively [5].

The effects of NPs on the SHCs of molten salt-based nanofluids have also been investigated [7–12]. Several studies have shown that the specific heat of liquid solar salt can be enhanced by doping NPs at minute concentrations. Ho and Pan measured the SHC of alumina nanoparticle-doped molten Hitec salt and identified an optimal concentration of 0.063 wt% of NPs that could yield a 19.9 % enhancement of SHC [7]. Dudda and Shin observed a 27 % increase in SHC for SiO_2_/solar salt nanofluid at 1 wt% [8]. Andreu-Cabedo et al. observed that the SHC of solar salt was enhanced by 25 % when the salt was mixed with 10 nm SiO_2_ NPs at 1 wt% [9]. Jo and Banerjee found that the SHCs of carbonate salts were significantly enhanced when dispersed with 0.1 wt% of graphite NPs. The maximum enhancements in the SHCs for the nanomaterials were 40 and 57 % in the solid and liquid phases, respectively [10].

Lu and Huang reported a 10 % decrease in the SHC of a solar salt nanofluid with a concentration of 4.6 wt% Al_2_O_3_ NPs [11]. Chieruzzi et al. reported a 7.5 % decrease in the SHC of solar salt after adding 0.5 wt% of SiO_2_–Al_2_O_3_ NPs. However, when the mass concentration of SiO_2_–Al_2_O_3_ NPs increased to 1.0 wt%, the SHC of solar salt increased by 22.5 %. Chieruzzi et al. also observed that the phase change temperature decreased by more than 8 °C when 1.0 wt% of SiO_2_–Al_2_O_3_ NPs was added to solar salt; meanwhile, the heat of fusion increased by more than 15 % [12]. In summary, doping NPs to liquid solvents can significantly modify the SHC of nanofluids, the melting point, and the heat of fusion. Furthermore, NP concentration plays an important role in modifying thermal storage characteristics.

In this work, the thermophysical properties of nanofluids based on solar salt and graphene nanoplatelets (GNPs) were investigated. The effects of GNP concentration on the SHC, melting temperature, and startup heat of nanofluids were discussed. The optimal GNP concentration was determined by conducting differential scanning calorimetry (DSC) analysis.

## Methods

### Preparation of Binary Molten Nitrate Salts

Molten salt was prepared through a static melting method. Four major processes were implemented for this method. First, sodium nitrate and potassium nitrate were dried in an oven at 150 °C for 24 h. Second, sodium nitrate and potassium nitrate were mixed at a ratio of 3:2. Third, the mixed nitrate salts were triturated in a ball mill at a rate of 500 r/min for 30 min. Fourth, the mixed nitrate salts were heated in a muffle furnace from room temperature to 350 °C at a rate of 10 °C/min, kept isothermal for 120 min, cooled to room temperature, and then triturated into powder again (Table 1).

**Table 1:** Chemicals used in the preparation of binary molten salts

Material	Purity	Melting point (°C)	Heat of fusion (J/g)	Density (g/cm^3^)
NaNO_3_	AR (≥99.0 %)	307	172	2.26
KNO_3_	GR (≥99.0 %)	333	266	2.11

### Nanofluid Synthesis Procedure

The previously prepared molten salt was utilized as the base material of nanofluids. Disc-shaped GNPs have diameters of 10–20 μm and thicknesses of 5–20 nm. According to literature [8, 13], the SHC of graphite is 1.66 J g^−1^ K^−1^, and the average SHC of solar salt within 250 and 420 °C is 1.48 J g^−1^ K^−1^.

**Fig. 1:**
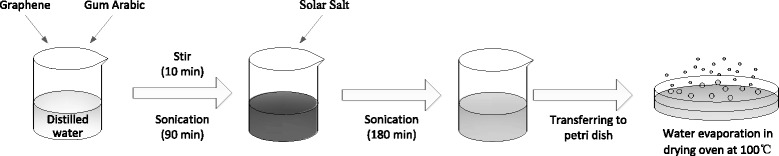
Schematic of the synthesis procedure for molten salt nanofluids

Solar salt-based nanofluids of different GNP concentrations within the range of 0.1–3.5 wt% were prepared. The nanofluid synthesis procedure, which is illustrated in Fig. 1, was conducted following the Jo and Banerjee method [10]. GNPs and gum arabic were initially added to distilled water. This suspension was stirred in a magnetic stirrer for 10 min and then subjected to sonication in a sonicator for 90 min. Then, the solar salt was added to the aqueous graphene nanofluid prepared in the previous step, and the aqueous salt-graphene nanofluid was sonicated again for 180 min to further disperse the GNPs. Afterward, the solution was heated in a drying oven at 100 °C to remove water. Lastly, the solution was evaporated in a petri dish to reduce the duration of complete evaporation and prevent the agglomeration of the GNPs.

### DSC

DSC measurements were performed with a Pyris Diamond DSC. The dried solar salt nanofluid was placed in a standard aluminum crucible and subjected to the following thermal cycle in nitrogen atmosphere. The temperature of the crucible was maintained at 150 °C for 5 min (to remove any absorbed water in the sample), increased from 150 to 450 °C at 20 °C/min, maintained at 450 °C for 5 min, and decreased from 450 to 150 °C at 20 °C/min. The first DSC measurement was discarded in the determination of SHC because the heat flux curve can shift as a result of non-thermal equilibrium. The other three cycles were run on each sample consecutively. Based on the measured heat flux differences, SHC was determined through the standard protocol established by the American Standard Test Method (ASTM E1269-2011) [14].

Similar DSC measurements were performed on the solar salt. The SHC in liquid phase, the melting temperatures, and the startup heat were compared with those of the solar salt nanofluids. Thus, the effect of NP concentrations can be evaluated.

## Results and Discussion

### Calorimetric Analysis

**Fig. 2:**
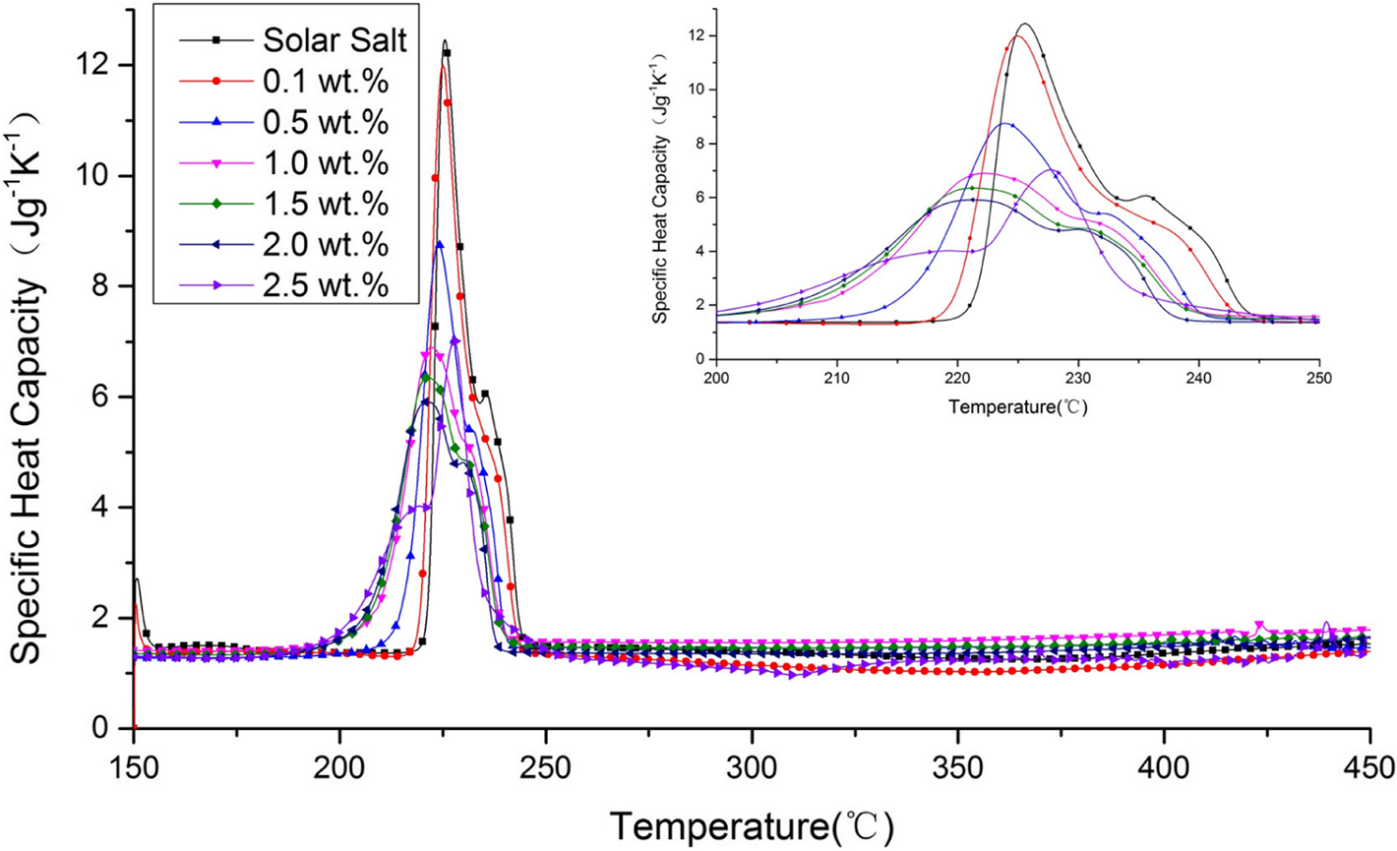
SHC versus temperature for molten salt and nanofluids with different GNP concentrations for temperature range from 150 to 450 °C. The *inset* shows the same variation within the temperature range from 200 to 250 °C

Figure 2 plots SHC versus temperature for molten salt and nanofluids with different GNP concentrations. As shown in Fig. 2, the different NP concentrations significantly changed the shape of the SHC curve. To analyze the effect of GNPs on the phase change process and heat absorbed by the thermal storage material at startup, the onset temperature, peak temperature, endset temperature, and startup heat of all the samples were obtained through DSC measurements. The results are listed in Table 2.

**Table 2:** Onset temperature, peak temperature, endset temperature, and startup heat of molten salt and nanofluids with different GNP concentrations

GNP concentration (wt%)	Onset temperature (°C)	Peak temperature (°C)	Endset temperature (°C)	Startup heat (J g^−1^)
–	221.6	225.6	243.7	238.58
0.1	219.9	225.0	242.8	232.33
0.5	216.4	223.9	239.7	208.56
1.0	211.2	222.2	239.0	216.98
1.5	208.9	221.2	238.8	208.13
2.0	207.9	220.9	237.3	199.08
2.5	201.0	227.6	234.4	186.37

According to the regulations of the International Conference on Thermal Analysis, the onset temperature is the intersection point of the baseline before the phase change process occurs and the inflectional tangent. The onset temperature is generally considered the melting point. However, from the point of view of application, the thermal storage material is not completely transformed into the liquid phase at the onset temperature. The temperature of a complete phase transformation is a certain temperature between the peak temperature and endset temperature. For this reason, the endset temperature is employed as the temperature of a complete phase transformation. The three characteristic temperatures are all useful in describing the melting behavior of thermal storage materials.

The DSC measurements showed that the onset temperature of the prepared solar salt was 221.6 °C, which is consistent with the literature value [1]. Except for the peak temperature of the nanofluid with 2.5 wt% NP concentration, the addition of GNPs reduced the onset temperature, peak temperature, and endset temperature of all samples. In particular, when 1.0 wt% NPs were added, the onset and endset temperatures of the nanofluid decreased by 10.4 °C (4.7 %) and 4.7 °C (1.9 %), respectively, compared with those of solar salt. When 2.5 wt% NPs were added, the onset and endset temperatures of the nanofluid decreased by 20.6 °C (9.3 %) and 9.3 °C (3.8 %), respectively. This result indicates that the phase transformation of nanofluids occurs at temperatures lower than those for molten salt. This phenomenon is an obvious advantage when nanofluids are utilized in solar plants.

Startup heat is the energy absorbed by a thermal storage material during the startup process of a power plant. Startup heat *ΔH* is defined as

1 $$ \varDelta H={\displaystyle {\int}_{T_{\mathrm{en}}}^{T_{\mathrm{en}\mathrm{dset}}}{C}_P(T)dT} $$

where *T*_en_ is the environment temperature and *C*_*P*_(*T*) is the SHC of molten salt.

The sensible heat of a thermal storage material at solid phase accounts for a small proportion of the startup heat. Adding NPs slightly changes the SHC of solar salt at solid phase. As shown in Fig. 2, the change is smaller in the solid phase than in the liquid phase. Accordingly, 160 °C was selected as the lower limit of the integral in Eq. (1).

As shown in Table 2, the addition of GNPs significantly reduced the startup heat of all samples. In general, the startup heat decreased with the GNP concentration except for the 0.5 wt%. When 1.0 wt% NPs were added, the startup heat of the nanofluid decreased by 21.60 J/g (9.1 %). When 2.5 wt% NPs were added, the startup heat of the nanofluid decreased by 52.21 J/g (21.9 %). The reduced startup heat could reduce the risk of local over-heating of molten salt.

In concentrated solar power systems, the sensible heat of molten salt at liquid state determines the heat capacity of the storage system. The operation temperature range of molten salt is usually from 250 to 450 °C. Table 3 shows the average value of SHC for each sample. The average value of SHC was calculated as follows:

**Table 3:** Average value of SHCs of molten salt and nanofluids from 250 to 450 °C

SHC (J g^–1^ K^–1^)	Solar salt	Concentration (wt%)					
		0.1	0.5	1.0	1.5	2.0	2.5
1st test	1.41	1.13	1.45	1.64	1.46	1.41	1.26
2nd test	1.35	1.18	1.49	1.57	1.56	1.43	1.48
3rd test	1.37	1.19	1.52	1.61	1.51	1.42	1.43
Average	1.38	1.17	1.49	1.61	1.51	1.42	1.39
Enhancement	–	−15.2 %	+7.9 %	+16.7 %	+9.4 %	+2.9 %	+0.7 %

2 $$ \overline{C_p}=\frac{{\displaystyle {\int}_{250}^{450}{C}_p(T)dT}}{450-250}. $$

As shown in Table 3, the average value of the SHC of solar salt was 1.38 J g^−1^ K^−1^. This value is relatively consistent with 1.48 J g^−1^ K^−1^, the average value from 250 to 420 °C according to literature [9]. Moreover, with the increase in GNP concentration, the SHC of nanofluid decreased initially (0.1 wt%). Then, it gradually increased and exceeded the SHC of solar salt (0.5 wt%). Next, it reached its maximum value (1 wt%), after which it decreased again (1.5, 2.0, and 2.5 wt%).

**Fig. 3:**
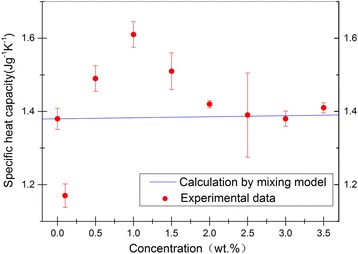
Average SHC versus particle concentration for molten salt and nanofluids (*symbols* experiment data, *line* model prediction). The *error bars* are set equal to one standard deviation

Figure 3 shows the variation in SHC with NP concentration. The experimental values of the heat capacity were plotted for all nanofluids and solar salt. According to the thermal equilibrium model (also called the mixture model), the effective SHC of the nanofluid, *C*_*p*,nf_, can be calculated by

3 $$ {C}_{p,\mathrm{n}\mathrm{f}}={w}_{\mathrm{np}}{C}_{p,\mathrm{n}\mathrm{p}}+{w}_s{C}_{p,s} $$

where *w* is the mass fraction and subscripts nf, np, and s refer to nanofluid, NP, and base molten salt, respectively.

Given that NP concentration was relatively small, the SHC values of nanofluids are nearly similar to those of solar salt, according to Eq. (3). By contrast, as shown in Table 3, when 1.0 wt% NPs were added, the SHC of the nanofluid was 1.63 J g^−1^ K^−1^. The SHC of the nanofluid was enhanced by 16.7 % with respect to that of the solar salt. When 0.1 wt% NPs were added, the SHC of the nanofluid was 1.17 J g^−1^ K^−1^; the SHC of the nanofluid decreased by 15.2 %. These results are contradictory to the predictions from Eq. (3). This difference implies that the thermal equilibrium model could not be applied to predict the SHC of nanofluids, and other mechanisms might have existed and led to the anomalous behavior.

As shown in Fig. 3, with the further increase in NP concentration, the experimental results tended to be close to the model predictions of Eq. (3). The deviation of the SHC values from the model predictions is less 2 % when the NP concentration is higher than 2.5 wt%. Therefore, we speculate that the nanoscale effects of NP gradually weakened with the further increase in NP concentration. Thus, the SHC value could be predicted by Eq. (3) by ignoring the nanoscale effects when the NP concentration was more than a certain value.

### Mechanism of SHC Change

**Fig. 4:**
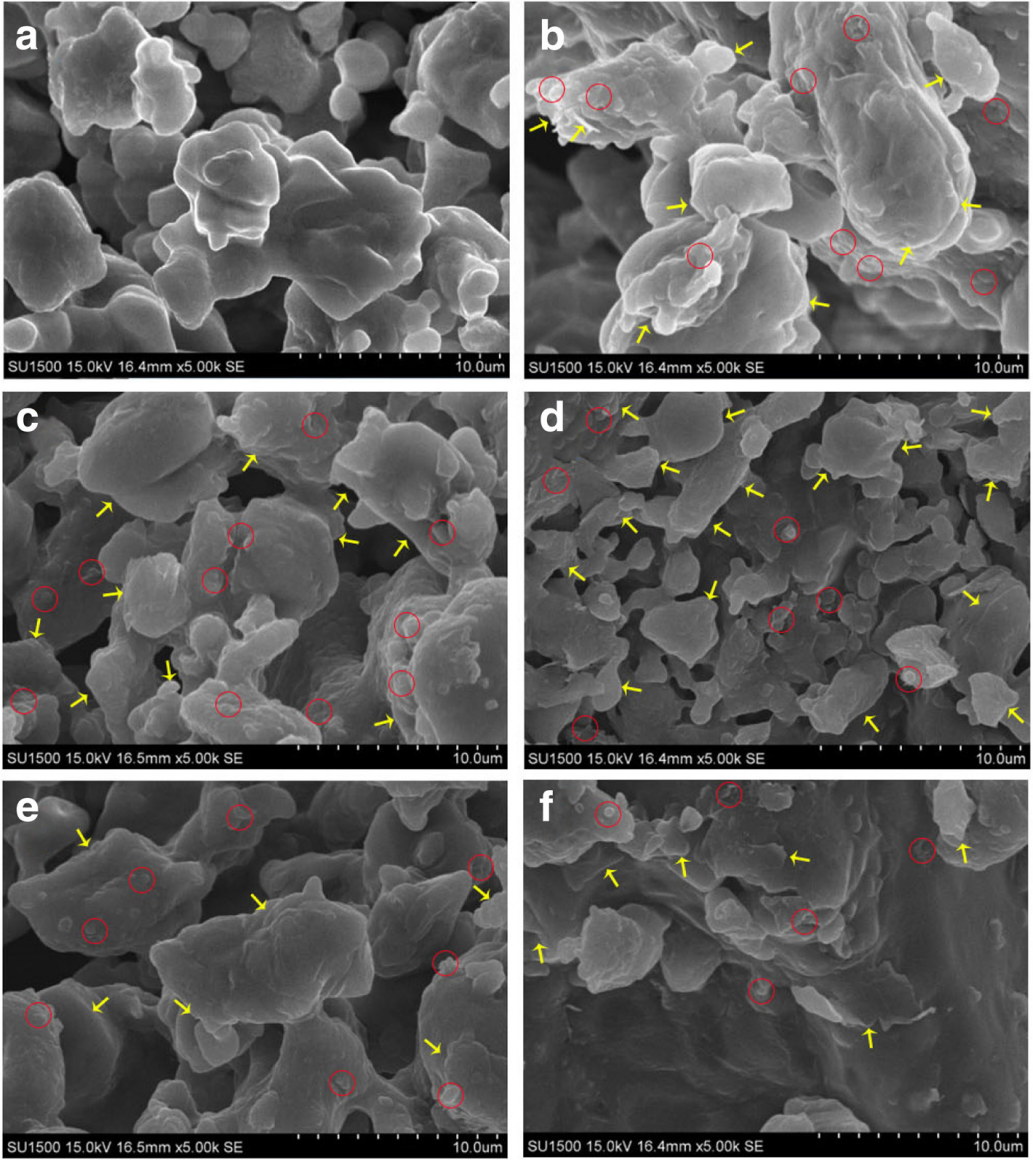
SEM pictures of molten salt and nanofluids with different particle concentrations: **a** solar salt, **b** 0.5 wt%, **c** 1.0 wt%, **d** 1.5 wt%, **e** 2.0 wt%, and **f** 2.5 wt% at ×5000 magnification. *Yellow arrows* indicate nanoplatelets, and *red circles* indicate clusters

The dispersion degree of particles in nanofluids is a key factor that affects the change in SHC. To analyze the dispersion degree of particles in nanofluids, scanning electron microscopy (SEM) characterization was performed. SEM pictures of the molten salt and GNPs are shown in Fig. 4. The tiny particles of pure molten salt are rounded and smooth-surfaced. On the contrary, some irregular stratification inside the tiny particles of nanofluids can be observed, and the particle surface is rough with protrusion of certain sheet-like structures. These structures are attributed to the added GNPs. We infer that the GNP particles were well dispersed in molten salt because large pieces of graphene platelets were not observed. With the increase in GNP concentration, the irregular stratification inside the tiny particles of nanofluids increased, and all the graphene platelets could be seen on the surface of the particles.

Thus far, studies on the mechanism of SHC change in molten salt-based nanofluids are rare, and most of them employ qualitative analyses rather than quantitative calculations. In general, the mechanisms for the change in SHC can be categorized into two groups. The first group attributes the mechanism to the solid–liquid interaction at the interface. Specific surface area is assumed to be the key factor. For example, the change in SHC is attributed to the ordered compressed liquid layer. The second group attributes the mechanism to the special structure formed in the nanofluid. For example, NP aggregates or clusters can introduce chain-like or net-like nanostructures inside the entire volume of nanofluids.

An ordered liquid layer could form on the surface of crystalline solids, and the thickness of this semi-solid layer is ~1 nm [15]. According to the proposed model based on a compressed liquid layer [16], the SHC of the compressed liquid layer should be negative if the SHC of nanofluid is less than those of pure molten salt and graphene. However, this deduction is illogical. Shin and Banerjee observed peculiar chain-like structures in the SEM images of nanofluids after a melting–solidification cycle [17]. However, the same structures were not found for the base molten salt prepared by the same procedure. These peculiar structures modified SHC and therefore contributed to the changes in the SHC of the entire nanofluids. Shin et al. proposed a modified model that considers the effect of these special structures as follows [17]:

4 $$ {C}_{p,\mathrm{n}\mathrm{f}}={w}_{\mathrm{np}}{C}_{p,\mathrm{n}\mathrm{p}}+{w}_{\mathrm{ns}}{C}_{p,\mathrm{n}\mathrm{s}}+\left({w}_s-{w}_{\mathrm{ns}}\right){C}_{p,s} $$

where *C*_*p*_ is the SHC and *w* is the mass fraction. The subscripts nf, np, ns, and s denote the thermophysical property values of the resultant nanofluid, NP, nanostructure, and base molten salt, respectively. Similar to the mixture model, the modified model assumes that the SHC of the nanofluid is the mass-weighted average value of the SHC of NPs, nanostructures, and base molten salt. However, quantifying *w*_ns_ and *C*_*p*_,_ns_ is extremely difficult at present.

The reason why the SHC of molten salt nanofluid decreased at 0.1 wt% of GNPs remains unclear. However, the trend of variation of the nanofluid SHCs observed in our work is similar to that observed by Chieruzzi et al. [12]. Chieruzzi et al. pointed out that the largest enhancement in SHC is generally obtained by adding 1 wt% of NPs, which agrees well with the optimal concentration for the GNP molten salt nanofluid. The same underlying mechanism might exist, and further investigation is required to obtain thorough understanding.

## Conclusions

The thermal storage properties of molten nitrate salt-based GNP nanofluids with different mass concentrations were studied. DSC was employed to obtain the thermophysical properties. SEM characterization was performed to investigate the dispersion degree of particles in nanofluids.

The DSC analysis showed that the addition of GNPs reduced the onset and endset temperatures of molten salt at a low temperature. In addition, the required heat in the process of startup decreased compared with that of the pure molten salt. The SHCs of the nanofluid samples at the liquid phase were significantly modified by the added GNPs. When *w*_*np*_ < 1 %, it reached a peak value at 1 wt%; after 1 %, SHC decreased with mass concentration when *w*_*np*_ > 1 %. In particular, the average SHC of nanofluid within the temperature range of 250-450 °C was enhanced by 16.7 at 1 wt% of GNPs. Furthermore, it was found that the measured SHC agreed with the prediction obtained from a thermal equilibrium model when *w*_*np*_ > 2.5 %. At the 1 wt% concentration, the onset temperature decreased by 10.4 °C, the endset temperature decreased by 4.7 °C, and the startup heat decreased by 9 %. The reason for the variation in SHC requires further investigation.

## Abbreviations

DSC, differential scanning calorimetry; GNP, graphene nanoplatelet; NP, nanoparticle; SEM, scanning electron microscope; SHC, specific heat capacity
